# Epidemic growth and Griffiths effects on an emergent network of excited atoms

**DOI:** 10.1038/s41467-020-20333-7

**Published:** 2021-01-04

**Authors:** T. M. Wintermantel, M. Buchhold, S. Shevate, M. Morgado, Y. Wang, G. Lochead, S. Diehl, S. Whitlock

**Affiliations:** 1 Physikalisches Institut, Universität Heidelberg, 69120 Heidelberg, Germany; 2 ISIS (UMR 7006), University of Strasbourg and CNRS, 67000 Strasbourg, France; 3 Institut für Theoretische Physik, Universität zu Köln, 50923 Cologne, Germany

**Keywords:** Ultracold gases, Phase transitions and critical phenomena, Complex networks

## Abstract

Whether it be physical, biological or social processes, complex systems exhibit dynamics that are exceedingly difficult to understand or predict from underlying principles. Here we report a striking correspondence between the excitation dynamics of a laser driven gas of Rydberg atoms and the spreading of diseases, which in turn opens up a controllable platform for studying non-equilibrium dynamics on complex networks. The competition between facilitated excitation and spontaneous decay results in sub-exponential growth of the excitation number, which is empirically observed in real epidemics. Based on this we develop a quantitative microscopic susceptible-infected-susceptible model which links the growth and final excitation density to the dynamics of an emergent heterogeneous network and rare active region effects associated to an extended Griffiths phase. This provides physical insights into the nature of non-equilibrium criticality in driven many-body systems and the mechanisms leading to non-universal power-laws in the dynamics of complex systems.

## Abstract (web summary)

The emergent excitation dynamics of an ultracold gas of Rydberg atoms exhibits features analogous to epidemic spreading on networks. Wintermantel et al. propose a controllable experimental system for studying network dynamics at the interface of mathematical models and real-world complex systems.

## Introduction

The dynamical behavior of an exceptionally diverse spectrum of real-world systems is governed by critical events and phenomena occurring on vastly different spatial and temporal scales. A disease outbreak, for example, can be very sensitive to the type of disease and the behavior of individuals, yet epidemics generically feature a characteristic time dependence^1^ that emerges from the connections within and between communities^2,3^. In studying these systems, complex networks provide a crucial layer of abstraction to bridge the behavior of individuals and the macroscopic consequences^4^. Accordingly, they have found applications not only in biology and the study of epidemics^2^, but also in informatics^5^, marketing^6^, finance^7^, and traffic flow^8^. An overarching challenge in these fields is to find general principles governing complex system dynamics and to pinpoint how apparent universal characteristics emerge from the underlying network structure.

**Fig. 1:**
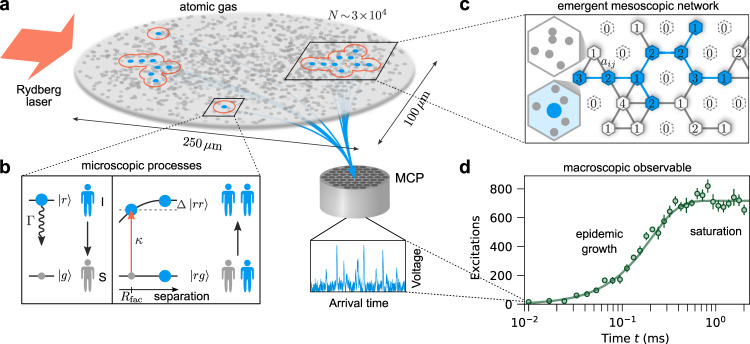
Physical system for studying epidemic growth and dynamics on complex networks. **a** Experiments are performed on a two-dimensional gas containing *N* ~ 3 × 10^4^ potassium atoms driven by an off-resonant laser field. The gas is initially prepared with a small number of seed excitations (blue disks), which then evolves according to the microscopic processes depicted in sub-figure b, giving rise to growing excitation clusters that spread throughout the system. After different exposure times *t*, the Rydberg atoms are field ionized and detected on a microchannel plate detector (MCP), where the incident ions create voltage spikes (blue trace). **b** Each atom can be treated as a two-level system with a ground state $\left|g\right\rangle$ (gray disks) and excited Rydberg state $\left|r\right\rangle$ (larger blue disks). Excited atoms can decay with rate Γ or facilitate additional excitations with rate *κ* at a characteristic distance *R*_fac_ (determined by the laser detuning Δ) analogous to the transmission of an infection. **c** The dynamics of this system can be described by a susceptible-infected-susceptible (SIS) model on an emergent heterogeneous network. Each node *i* represents a discrete cell of the coarse-grained system, which can be infected (with excitation, blue) or susceptible (without excitation, white) and is connected to neighboring nodes according to the adjacency matrix *a*_*ij*_. The infection probability of each node is weighted by the number of atoms in that cell that can undergo facilitated excitation *N*_*i*_ (indicated by the numerical labels on each node). Disconnected nodes with *N*_*i*_ = 0, corresponding to vacant cells, are depicted with dashed lines. **d** Exemplary data and numerical simulations (solid line) showing two different stages of dynamics: rapid growth followed by saturation. Error bars represent the standard error of the mean over typically 16 experiment repetitions.

In this work, we address this challenge using a highly controllable complex system that consists of a trapped ultracold atomic gas continuously driven to strongly interacting Rydberg states by an off-resonant laser field (Fig. 1). Our main findings include: first, the rapid growth of excitations driven by a competition between microscopic facilitated excitation and decay processes (playing the role of the transmission of an infection and recovery, respectively). The observed dynamics follow a power-law time dependence that parallels that which is empirically observed in real-world epidemics, providing a powerful demonstration of universality reaching beyond physics. Second, a full description and interpretation of the experiment in terms of an emergent susceptible-infected-susceptible (SIS) network linking the observed macroscopic dynamics to the microscopic physics. Third, the unexpected presence of rare region effects and a dynamical Griffiths phase^9–11^ associated with the emergent network structure, which gives rise to critical dynamics over an extended parameter regime and explains the appearance of power-law growth and relaxation, but with non-universal exponents.

## Results

### Microscopic ingredients for an epidemic

The microscopic processes governing the dynamics of ultracold atoms driven to Rydberg states by an off-resonant laser field, shown in Fig. 1a, b, bear close similarities to those in epidemics^12^. Each atom can be considered as a two-level system consisting of the atomic ground state (gray disks, healthy) and an excited Rydberg state (blue disks, infected). An excited atom can spontaneously decay (recovery, with rate Γ), or it can facilitate the excitation of other atoms (transmission of the infection, with rate *κ*) that satisfy certain constraints linked to their positions and velocities. This results in rapid spreading of the excitations through the gas (depicted by growing excitation clusters in Fig. 1a)^13–17^.

Our experimental studies start from an ultracold thermal gas of 3 × 10^4^ potassium-39 atoms in their ground state $\left|g\right\rangle =4{s}_{1/2}$, which are held in a two-dimensional optical trap with a peak atomic density *n*_2*D*_(*x* = *y* = 0) = 0.76 μm^−2^ (Fig. 1a) and *e*^−1/2^ Gaussian widths of the atomic distribution of *σ*_*x*_ = 125 μm and *σ*_*y*_ = 50 μm. To trigger the dynamics at *t* = 0 we apply a low-intensity laser pulse tuned in resonance with the $\left|g\right\rangle \to \left|r\right\rangle =66{p}_{1/2}$ transition for a duration of 4 μs. This produces around eight seed excitations at random positions within the gas. The laser is then suddenly detuned from resonance by Δ = −30 MHz and adjusted in intensity. This makes it possible to facilitate secondary excitations at an average distance *R*_fac_ = 3.5 μm (illustrated by red circles), corresponding to the distance where the dominant Rydberg pair-state energy compensates the laser detuning. The two-body facilitation rate *κ* is proportional to the laser intensity which can be tuned over a wide range. This can be understood as an effective rate averaged over the different excitation channels corresponding to many Rydberg pair-state interaction potentials. We therefore experimentally calibrate *κ* based on the characteristic doubling time measured at very early times (see Methods). For the following measurements we choose different values of *κ* ranging from 3.3 to 10 kHz. Additionally, Rydberg excitations spontaneously decay after a characteristic lifetime *τ* = (2*π*Γ)^−1^ with a calculated rate Γ = 0.84 kHz (including black-body decay). There is also a strong dephasing of the ground-Rydberg transition with estimated rate *γ*_de_ ≳ 200 kHz. This implies that in the experimentally relevant regime *t* ≳ 1 μs, the dynamics can be well described by classical transition rates. Spontaneous (off-resonant) excitation events are very rare, observed in an independent experiment without triggered seed excitations to be  ≲1 kHz integrated over the whole cloud. To observe the system we measure the total number of Rydberg excitations present in the gas using field ionization and a microchannel plate (MCP) detector for different exposure times *t* up to 2 ms. Although each atom is identical and its evolution is captured by these simple excitation rules, the competition between facilitated excitation and decay gives rise to complex dynamical phases and evolution^18–23^. However, the full many-body system is even more complex: 3 × 10^4^ multilevel atoms moving in space with random positions and velocities while interacting with the laser field and each other, which makes it challenging to connect the microscopic physics to the macroscopic excitation dynamics^22,24,25^.

### Observation of epidemic growth

To exemplify the analogy to epidemics, in Fig. 1d we present data for *κ* = 10 kHz showing different stages of the dynamics. Immediately following the seed excitation pulse we observe a period of very fast growth of the Rydberg excitation number, that is, within the Rydberg state lifetime the excitation number increases from its initial value to more than 400, corresponding to more than five doublings in 0.19 ms. At around *t* ≈ 0.5 ms, after the initial growth stage, the system saturates with a high constant excitation number (i.e., an endemic state). However, the saturation value is still significantly lower than the estimated maximum number of excitations that can fit in the system ≳2000 assuming an inter-Rydberg spacing of ~*R*_fac_. On even longer timescales than those studied here (≳10 ms), the system should eventually relax back to an absorbing or self-organized critical state due to the gradual depletion of particles^23,26^.

The growth phase of many real epidemics is observed to follow a characteristic power-law dependence described by the phenomenological generalized-growth model (GGM)^1^,

1 $$C^{\prime} (t)=r{C}^{p}(t).$$

This describes a relation between incidence rate $C^{\prime}$ and cumulative number of infections $C=\mathop{\int}\nolimits_{0}^{t}C^{\prime} (t^{\prime} )dt^{\prime}$, where *r* is the growth rate at early times and *p* is the “deceleration of growth” which is an important parameter in classifying epidemics^1^. Exponential growth in time is characterized by *p* = 1, while *p* < 1 corresponds to power-law growth  ∝ *t*^*η*^ with *η* = *p*/(1 − *p*).

**Fig. 2:**
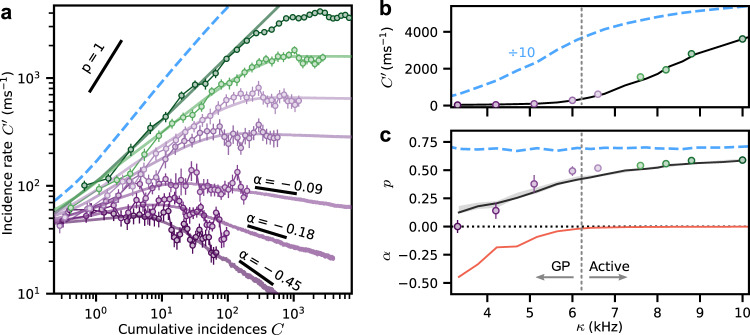
Rydberg excitation incidence curves for different facilitation rates showing power-law growth and Griffiths effects. **a** Incidence rate $C^{\prime}$ versus cumulative incidences *C* for different facilitation rates *κ* = {3.3, 4.2, 5.1, 6.0, 6.6, 7.6, 10} kHz (from purple to green). Error bars show the standard error of the mean over typically 16 repetitions of the experiment. The straight dark green line is a power-law fit to a representative dataset yielding the exponent *p* = 0.59(1). The solid curves are from the simulations of the SIS model on a heterogeneous network and the blue dashed line is a corresponding simulation for a locally homogeneous network for *κ* = 10 kHz and a comparable system size which gives *p* ≈ 0.67. **b** Transition from a subcritical state ($C^{\prime} \approx 0$) to an active state ($C^{\prime} \,> \, 0$) at late times *t* = 2 ms as a function of *κ*. The solid black curve and the dashed blue curve (scaled by a factor of 10 for visual comparison) show simulations of the heterogeneous and locally homogeneous network models, respectively, which exhibit different thresholds and incidence rates. **c** Characterization of the deceleration of growth parameter *p* (experimental data points and simulations as a black line) and power-law relaxation exponent *α* (orange line, numerical simulations only) versus *κ*. The vertical dashed line indicates the cross-over point between the Griffiths phase (GP) and the active phase. Uncertainties computed from the standard deviation over 100 bootstrap resamplings are shown as error bars except where they are smaller than the data points.

In Fig. 2a we represent the data from Fig. 1d in terms of $C^{\prime}$ (instantaneous number of excitations divided by their lifetime *τ* = (2*π*Γ)^−1^) against its time integral *C*, shown by the darkest green data points. This clearly shows that the incidence rate follows the GGM over several decades (evidenced by a straight line on a double logarithmic scale) with a deceleration of growth parameter *p* = 0.59(1) that is comparable to empirical observations of real epidemics^1^. In fact power-law growth with varying exponents *p* < 0.6 is a general feature of the system dynamics, as seen in Fig. 2a for different *κ* values from 3.3 to 10 kHz (depicted with different colors), together with the corresponding *p* values plotted in Fig. 2c as determined from fits to the initial growth stage (see Supplementary Note 1). This is to be contrasted with exponential growth (*p* = 1, solid black line with a steeper slope). We also point out that each curve saturates at a different *κ*-dependent value, with some curves showing evidence for slow relaxation back toward zero incidences (the lowest three curves in Fig. 2a). In the study of epidemics, power-law growth with *p* < 1 is commonly associated with a few underlying mechanisms, most prominently spatial constraints and heterogeneity in the underlying network structure^1^. In the following we use this insight to develop a spatial network model which quantitatively describes the experimental observations and can be directly linked to the microscopic details of the system, something that is rarely possible for real epidemics.

### Emergent heterogeneous network

To explain the experimental observations we develop a physically motivated SIS network model. We assume that the two-dimensional gas can be subdivided into cells that represent nodes of a network (Fig. 1c). Each cell *i* can either be in a susceptible state (absence of Rydberg excitation, *I*_*i*_ = 0) or infected (one Rydberg excitation, *I*_*i*_ = 1), and contains a certain number of particles *N*_*i*_ that can be excited. Vacant cells with *N*_*i*_ = 0 (and hence also *I*_*i*_ = 0) translate to unconnected, missing nodes. The probability for a given node *i* to become infected is described by the following stochastic master equation^2^

2 $$\frac{dE[{I}_{i}(t)]}{dt}=E\left[-\Gamma {I}_{i}(t)+\kappa {N}_{i}\left(1-{I}_{i}(t)\right)\sum _{j}{a}_{ij}{I}_{j}(t)\right],$$

where *E*[⋅] denotes the expectation value. The node weights *N*_*i*_ and the adjacency matrix *a*_*ij*_ together determine the probability for transmission of an infection from cell *j* to *i*. In the special case ${N}_{i}={\rm{const.}},{a}_{ij}=1$, this reduces to the well-studied homogeneous compartmental model^2^ that exhibits exponential growth. For ${N}_{i}={\rm{const.}}$ and assuming a regular lattice with nearest-neighbor transmission, this model is equivalent to directed percolation concerning its universal properties^27^. However, spatially structured adjacency matrices can give rise to more complex spatio-temporal evolution^28^.

To define the adjacency matrix entries *a*_*ij*_ we coarse grain our system into hexagonal cells (each with area $\sim \!{R}_{{\rm{fac}}}^{2}$), corresponding to a triangular network of nodes, where *a*_*ij*_ = 1 for each of the six nearest neighbors *j* to each node *i* and *a*_*ij*_ = 0 for all other nodes *j*. This is motivated by the fact that hexagonal packing provides the densest possible tiling of strongly interacting Rydberg excitations in two-dimensional space^29^, although the underlying atomic gas has no such apparent structure. The *N*_*i*_ are sampled from a Poissonian distribution with a spatially dependent mean ${\mu }_{i}=\epsilon (\kappa ){n}_{2{\rm{d}}}({x}_{i},{y}_{i}){R}_{{\rm{fac}}}^{2}$, where *ϵ*(*κ*) < 1 is the accessible phase space fraction for facilitated excitation (a free parameter, elaborated on below) and *n*_2d_(*x*_*i*_, *y*_*i*_) is the two-dimensional Gaussian density distribution of atoms in the trap. Thus, Eq. (2) describes a heterogeneous network where each node has a (spatially) fluctuating weighted degree *s*_*i*_ = ∑_*j*_*a*_*ij*_*N*_*j*_ with a mean and variance approximately equal to 6*μ*_*i*_.

To numerically simulate this model we solve Eq. (2) using a Monte-Carlo approach^30^. In each time step we compute the transition rate for each node *R*_*i*_ = *κN*_*i*_(1 − *I*_*i*_)∑_*j*_*a*_*ij*_*I*_*j*_ + Γ*I*_*i*_. One node *m* is then picked at random according to the weights *R*_*i*_ and its state is flipped *I*_*m*_ → 1 − *I*_*m*_. The timestep is computed according to $dt=-{\mathrm{ln}}\,(X)/(2\pi {\sum }_{i}R_{i})$, where $\mathrm{ln}\,$ is the natural logarithm and *X* is sampled from a uniform distribution on [0, 1). For the initial state we consider a fixed number of ∑_*i*_*I*_*i*_(*t* = 0) = 8 seed excitations randomly distributed among the nodes according to their weights *N*_*i*_.

The numerical simulations, shown as solid curves in Figs. 1d and 2, are in excellent agreement with the experimental observations. Importantly, they fully reproduce the fast power-law growth with *p* < 0.6, the different plateau heights, and even the late-time relaxation as a function of *κ*. The only free parameter in the model is *ϵ*(*κ*) which is adjusted for each curve and is found to be a monotonically increasing function of *κ* with 0.02 < *ϵ*(*κ*) < 0.1 over the explored parameter range. This parameter directly controls the network structure, that is, for *κ* = 10 kHz the network consists of *M* ≈ 2300 nodes with *N*_*i*_ > 0 and the local *s*_*i*_ follow approximately Poissonian distributions with 〈*s*_*i*_〉 = var(*s*_*i*_) ≤ 5.3 (maximal at trap center) while for *κ* = 3.3 kHz, *M* ≈ 660, and 〈*s*_*i*_〉 = var(*s*_*i*_) ≤ 1.3 (see Supplementary Note 2). For comparison, the dashed blue lines in Fig. 2 show comparable simulations with *N*_*i*_ = *μ*_*i*_, that is, corresponding to a locally homogeneous network with the same average node degree. These homogeneous network simulations show faster initial growth, constant *p* values  ≈ 0.7, higher plateaus saturating at the system size limit, and a dramatic shift of the critical point to lower *κ* values, which are inconsistent with the experimental data. The good agreement between experiment and heterogeneous network simulations demonstrates that the emergent macroscopic dynamics of the system crucially depend on the weighted node degree distributions and heterogeneity controlled by the atomic density and the parameter *ϵ*(*κ*).

The heterogeneous spatial network model described by Eq. (2) provides an accurate and computationally efficient coarse-grained description of the physical system and its dynamics involving just a few microscopically controlled parameters. The importance of heterogeneity is particularly surprising since atomic motion could be expected to quickly wash out the effects of spatial disorder (the characteristic thermal velocity corresponding to the gas temperature at 20 μK is *v*_*th*_ = 65 μm/ms ≈3.5*R*_fac_/*τ*). Our findings can be explained by assuming that the facilitation constraint depends on both the relative positions and velocities of the atoms. Taking into account the Landau–Zener transition probability for moving atoms confirms that only atom pairs with small relative velocities *v*_LZ_ ≲ 1 μm/ms contribute to the spreading of facilitated excitations^22^. This provides a qualitative explanation for the inferred *ϵ*(*κ*) ≪ 1 and its approximate *κ* dependence (due to the intensity dependence of *v*_LZ_) (see Methods section). It also sets the timescale for diffusion in phase space longer than the duration of our observations  ≳ 2 ms. Thus, spatial constraints and (effectively static) heterogeneity can be understood as properties of an emergent network structure that is dynamically formed while the laser coupling is on (see also ref. ^31^ for a related interpretation of excitation dynamics on smaller preformed emergent lattices).

Spatial disorder is known to play a very important role in condensed matter systems, giving rise to new many-body phases, localization effects, and glassy behavior^32^. There is still much to be explored concerning analogous effects of disorder and heterogeneity on non-equilibrium processes on networks. One key theoretical finding however is the emergence of an exotic Griffiths phase^9–11^, expected to replace the singular critical point between the subcritical and active phase by an extended critical phase. The dynamics in the Griffiths phase can be understood in terms of the dynamics of rare supercritical clusters (*κN*_*i*_ ≳ Γ for all sites *i* of the cluster), surrounded by subcritical regions. For a Poissonian degree distribution the probability for a seed to land on a supercritical cluster of *M*_clust_ nodes is $p({M}_{{\rm{clust}}}) \sim \exp (-x{M}_{{\rm{clust}}})$, where *x* is a positive function of *ϵ*_*i*_. The excitation number in such clusters will grow initially up to a typical lifetime $\tau ({M}_{{\rm{clust}}}) \sim \exp (y{M}_{{\rm{clust}}})$, for some positive constant *y*, after which it will decay due to rare fluctuations^11^. This yields an incidence $C^{\prime} (t)={\sum }_{{M}_{{\rm{clust}}}}p({M}_{{\rm{clust}}})\exp (-t/\tau ({M}_{{\rm{clust}}})) \sim {t}^{\alpha }$ with a non-universal decay exponent *α* = −*x*/*y* (dependent on *ϵ*_*i*_). This can lead to slow relaxation and strong modifications to the non-equilibrium critical properties (e.g., power-law correlations with continuously varying exponents^10^).

Such Griffiths effects provide a natural explanation for several of our experimental observations. First of all, the relatively short time for each curve to reach the plateau and the strong *κ* dependence of the plateau heights are compatible with the presence of rare regions with an above-average infection rate that span only a fraction of the entire system, controlled by the disorder strength entering via *ϵ*(*κ*). This also explains the sizable shift of the critical point between subcritical (${C}^{\prime}\approx 0$) and active (${C}^{\prime}\,> \, 0$) phases to higher values of *κ* as compared to the expectation for a locally homogeneous system seen in Fig. 2b. Finally, we point out the slow relaxation of the subcritical curves in Fig. 2a. These curves are compatible with power-law decays with disorder dependent relaxation exponents *α* < 0, which is the defining characteristic of the Griffiths phase^11^. While these experiments were limited to relatively short times <2 ms (to minmize the impact of particle loss), the numerical simulations confirm power-law relaxation over two orders of magnitude in time, depicted by solid lines in Fig. 2a. These relaxation exponents *α* were obtained from fitting an extended GGM (see Methods Eq. (3)) to the data, with corresponding *α* ≤ 0 values shown in Fig. 2c. On this basis we find that power-law growth (with 0.5 ≤ *p* ≤ 0.6) is associated with the transition from a Griffiths phase to an active phase (for *κ* > 6 kHz coinciding with *α* ≈ 0), whereas the absorbing state phase transition without disorder should occur for *κ* ≲ Γ^11^.

## Discussion

This work highlights a controllable physical platform for experimental network science situated at the interface between simplified numerical models and empirical observations of real-world complex dynamical phenomena. Ultracold atoms provide the means to introduce and control different types of reaction-diffusion processes as studied here, but also to realize different types of spatial networks by structuring the trapping fields^33^ and to access the full spatio-temporal evolution of the system^29^. This would allow for in-depth investigations of the phase structure and critical properties with varying disorder strength and different network geometries. Our discovery that the growth dynamics of a driven-dissipative atomic gas is described by an emergent heterogeneous network that is relatively robust to particle motion suggests that similar effects could also be observable in noisy room temperature environments^16,26^. Thus, heterogeneous network dynamics and Griffiths effects may arise naturally in very different non-equilibrium systems, having important implications, for example, in understanding non-equilibrium criticality without fine tuning^23,26,34^ and for finding effective strategies for controlling dynamics on complex networks^35^. Future experiments could also investigate the quantum contact process^12,36–38^ and quantum analogs of the Griffiths phase on heterogeneous networks^39^.

## Methods

### Experimental sequence and calibration of parameters

The experimental procedure to observe Rydberg excitation growth consists of three main steps, during which we keep the optical trap on. Initially a small number of seed excitations are prepared at random positions in the gas. For this we keep the laser frequency fixed at Δ = −30 MHz below the zero-field resonance and briefly applying an electric field of 0.28 V/cm for 4 μs, exploiting the DC Stark effect to tune the atoms into resonance. The laser is then momentarily switched off for 6 μs to ensure the electric field is fully off before starting the off-resonant driving. Next we apply the off-resonant laser field which causes rapid growth of the number of excitations in the gas. We calibrate the single-atom facilitation rate *κ* against a measurement of the initial growth rate *r* = 27(8) kHz, measured for high intensity and very short times *t* ≪ *τ* where many-body effects can be safely neglected. This is then divided by an estimate of the (cloud averaged) mean number of particles that meet the facilitation condition $\bar{\mu }=2.7$ assuming each seed excitation is isolated. The latter is estimated from the detailed experiment-theory comparison to the spatial SIS model presented in the manuscript. After a variable exposure time *t* we measure the total number of excitations in the gas. For this we switch on a large electric field to ionize the Rydberg states and guide the ions onto an MCP detector. The conversion factor from integrated MCP voltage to the number of Rydberg excitations is calibrated against an independent absorption measurement of the number of particles removed from the gas after a long exposure assuming each Rydberg excited atom is eventually lost from the trap with rate Γ.

### Extended generalized-growth model (GGM)

To extract both the growth and relaxation parameters from these data, we extend the GGM to allow for different exponents in the growth and relaxation phases

3 $$C^{\prime} (t)=r{C}^{p}(t){\left[1+{\left(\frac{C(t)}{K}\right)}^{\frac{p-\alpha }{\beta }}\right]}^{-\beta }.$$

In this equation *p* is the deceleration of growth parameter, *α* is the power-law exponent for the late-time recovery phase. *K* and *β* determine the location and sharpness of the crossover. Curves with *α* < 0 will eventually recover (i.e., number of excitations decreases to zero) while *α* = 0 describes an endemic state. The endemic state is characterized by a constant number of excitations $C^{\prime} =r{K}^{p}$. The number of excitations at the crossover point (*C* = *K*) is $C^{\prime} =r{K}^{p}/{2}^{\beta }$.

### Landau–Zener probability for facilitated excitation

By comparing the SIS network simulations to the data, we infer that the fraction of atoms that participate in the excitation dynamics is relatively small. This is quantified by the fitted *ϵ*(*κ*) values that vary between 0.023 and 0.094 (for *κ* = 3.3 kHz and *κ* = 10 kHz, respectively). These small values of *ϵ* and the approximate *κ* dependence can be explained by the velocity dependence of the Landau–Zener transition probability, which restricts facilitation to atoms with small relative velocities *v* ≲ *v*_LZ_ ≪ *v*_th_ (for a related calculations see Appendix E in ref. ^22^). The Landau–Zener velocity can be expressed as ${v}_{{\rm{LZ}}}={\pi }^{2}{\Omega }^{2}/\dot{V}$, where Ω is the light-matter coupling strength and $\dot{V}$ is the slope of the Rydberg-Rydberg interaction potential evaluated at the facilitation radius^22^. *ϵ* can be understood as the number of atoms that can be facilitated in a neighboring cell within the Rydberg state lifetime divided by the mean number of atoms in each cell ${n}_{2d}{R}_{fac}^{2}$. The flux of atoms passing through a 1/6 segment of the facilitation shell is Φ = *πR*_*fac*_*n*_2*d*_*v*_*th*_/3. However, only a fraction of these atoms ${f}_{v}\approx {v}_{{\rm{LZ}}}/\sqrt{\pi }{v}_{{\rm{th}}}$ fulfill the Landau–Zener condition with relative velocity ∣*v*∣ < *v*_LZ_. Combining the above gives $\epsilon =\Phi \tau {f}_{v}/{n}_{2d}{R}_{fac}^{2}\approx \sqrt{\pi }\tau {v}_{{\rm{LZ}}}/(3{R}_{fac})$.

For realistic experimental parameters *R*_fac_ = 3.5 μm, $\dot{V}=1\times 1{0}^{5}$ kHz μm^−1^ (ref. ^40^) and Ω ~100 kHz, we find *v*_LZ_ = 1 μm/ms. This is small compared to the thermal velocity *v*_th_ = 65 μm/ms for *T* = 20 μK. Inserting these parameters into the expression above for the phase space fraction yield $\epsilon =0.03{\left(\frac{\Omega }{100{\rm{kHz}}}\right)}^{2}$. This simple estimate falls within the range of values inferred from the experiment-theory comparison, even though it still does not account for all the microscopic experimental details, for example, multiple excitation resonances associated with Zeeman substructure or possible mechanical forces between the atoms.

## Supplementary information

Supplementary Information

Peer Review File

## Data Availability

The data that support the findings of this study are available from the corresponding author upon reasonable request.

## Code availability

The code to produce the simulation data that support the findings of this study is available from the corresponding author upon reasonable request.
