# Supplementary material for: Epidemic growth and Griffiths effects on an emergent network of excited atoms
Source: Nat Commun. 2021 Jan 4;12:103. doi: 10.1038/s41467-020-20333-7 (PMC7782709; doi:10.1038/s41467-020-20333-7)
Supplement: Supplementary file 1 — Supplementary Information [file 41467_2020_20333_MOESM1_ESM.pdf]

Supplementary Information for

Epidemic growth and Griffiths effects on an emergent network of excited atoms

T. M. Wintermantel[1,2], M. Buchhold[3], S. Shevate[2], M. Morgado[2], Y. Wang[2], G. Lochead[2], S. Diehl[3], S. Whitlock[2*]

[1]Physikalisches Institut, Universität Heidelberg, 69120 Heidelberg, Germany
[2]ISIS (UMR 7006), University of Strasbourg and CNRS, 67000 Strasbourg, France
[3]Institut für Theoretische Physik, Universität zu Köln, 50923 Cologne, Germany

[*]Corresponding author. E-mail: whitlock@unistra.fr.

November 6, 2020

Supplementary Note 1: Extracting the growth and relaxation parameters

Supplementary Figure 1a shows the complete experimental data-set up to 2 ms off-resonant laser excitation and for facilitation rate values of $\kappa = \{3.3, 4.2, 5.1, 6.0, 6.6,\ 7.6, 8.2, 8.8, 10\}$ kHz, color-coded from purple to green. The solid lines are the best fit numerical simulation results of the susceptible-infected-susceptible (SIS) network model as described in the manuscript using $\epsilon(\kappa) = \{0.023, 0.027, 0.037, 0.056, 0.067, 0.078, 0.083, 0.088, 0.094\}$.

To extract the growth and relaxation parameters from this data, we fit the extended generalized growth model (see Eq. (3) in the Methods section of the manuscript). Supplementary Figure 1b shows the incidence rate C' against its time integral C for the full experimental data

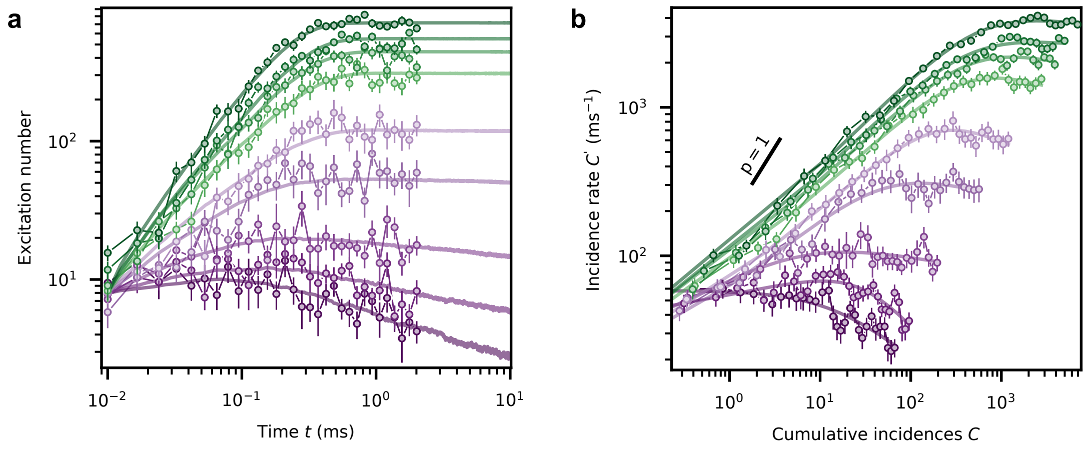

Supplementary Figure 1: **Extracting the growth and relaxation parameters on the full data set.** **a** Number of instantaneous excitations as a function of excitation time t showing all the raw experimental data (disks), where the colors from purple to green correspond to increasing facilitation rates κ values of $\{3.3, 4.2, 5.1, 6.0, 6.6, 7.6, 8.2, 8.8, 10\}$ kHz. The solid lines result from simulations using the described network SIS model. **b** Incidence rate C' versus cumulative incidences C. The data points (disks) are obtained from the data shown in sub-figure a. The solid colored lines show the fit results of the extended generalized growth model (see text for more details). The solid black line corresponds to exponential growth with $p = 1$. The error bars of the measurement data (disks) represent the standard error of the mean over typically 16 experimental runs.

set and the fits of the extended generalized growth model (solid lines). We observe good agreement between the data and the fit curves. The p parameters corresponding to these fit results are presented in Fig. 2c in the manuscript.

Supplementary Note 2: Characterization of the emergent network structure

Finally we present a statistical analysis of the emergent network structure inferred from the SIS simulations that reproduce the experimental data. In Supplementary Figure 2a,b we present the weighted degree distributions for a subcritical and supercritical network for $\epsilon(\kappa = 3.3\,\text{kHz}) = 0.023$ and $\epsilon(\kappa = 10\,\text{kHz}) = 0.094$ respectively, obtained from averaging over 1000 individual

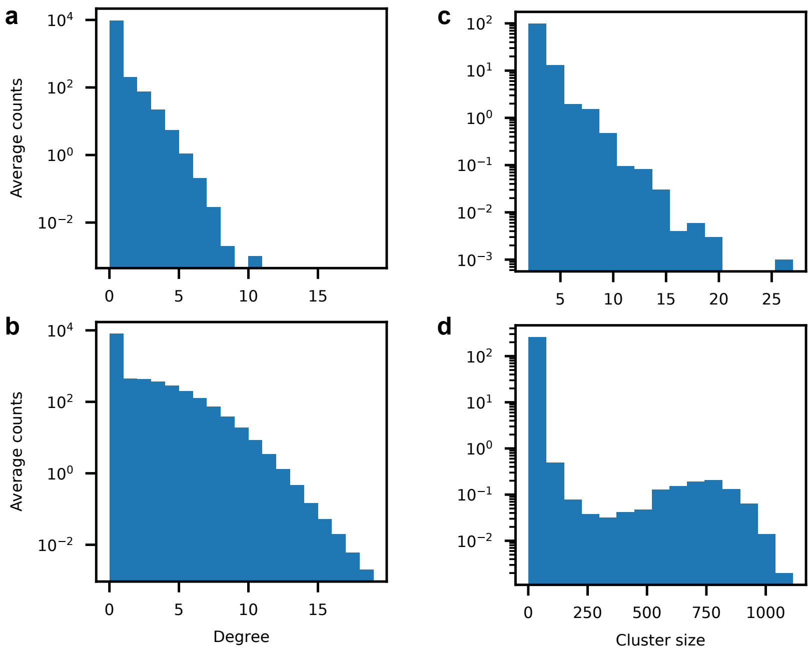

Supplementary Figure 2: **Characterization of the underlying structure of the network used in the SIS model simulations.** Weighted degree distributions for **a** $\kappa = 3.3\,$kHz (subcritical) and **b** $\kappa = 10\,$kHz (supercritical). Cluster size distribution for **c** $\kappa = 3.3\,$kHz and **d** $\kappa = 10\,$kHz.

network preparations. As for the simulations presented in the main paper, the system size is limited to a maximum of 10000 nodes. The weighted degree distributions are qualitatively similar, following an approximately Gaussian dependence as evidenced by parabolic shape on a semilog scale. However these networks are very different, as evidenced by the connected component or cluster size distribution. A cluster is defined as a set of nodes where each node one can reach any other node via edges, and the cluster size is the number of nodes in this set. Starting from a single seed excitation, the maximum number of excitations will be constrained by the size of its cluster. Supplementary Figure 2c shows that the cluster size distribution for the subcritical network with $\kappa = 3.3\,$kHz follows an approximately exponential dependence, i.e. large connected clusters are exponentially rare, consistent with the phenomenology of a Griffiths phase. In contrast, the supercritical network with $\kappa = 10\,$kHz exhibits much larger connected clusters with a peak in the cluster size distribution around 750 (Supplementary Figure 2d).
