# Supplementary material for: Epidemic growth and Griffiths effects on an emergent network of excited atoms
Source: Nat Commun. 2021 Jan 4;12:103. doi: 10.1038/s41467-020-20333-7 (PMC7782709; doi:10.1038/s41467-020-20333-7)
Supplement: Supplementary file 2 — Peer Review File [file 41467_2020_20333_MOESM2_ESM.pdf]

Reviewer #1 (Remarks to the Author):

The manuscript by Wintermantel et al. shows an interesting mapping of an experimental study of ultracold gas of Rydberg atoms onto an epidemic growth model on complex networks. In particular, the authors show that the observed excitation behavior is identical to the sub-critical dynamical scaling of the SIS model in an extended, so called, Griffiths Phase (GP).

Such Griffiths Phases have recently have been shown to appear in various network models of non-equilibrium models as the consequence of heterogeneity.
However, direct observation of GP has not been so easy, due to the size limitations of experiments, hindering to see clear scaling regions, which cause rounding and cut-off in time. Therefore the application of SIS on a real solid state system is significant to the field of condensed matters and related fields, like non-equilibrium statistical physics.

Here we one arrives at a question how well the classical SIS model can describe an ultracold gas, which is a quantum system. I miss a discussion about it. Could the authors also determine the critical behavior perhaps ? At line 189 "disorder-free absorbing state phase transition" is claimed for \kappa < \Gamma. What is the expectation for the universal critical point scaling : classical SIS, with quenched disorder or a quantum SIS with quenched disorder? The the quantum contact process, which presumably shares the universality of quantum SIS has just recently been explored. I think a paragraph on this would be desirable to compare to the established literature.

The work supports the conclusions and claims, based on numeric data of the excitation measurements and SIS simulations, however for claiming a real GP, defined in the thermodynamic limit, a system size independence should also been shown. In the lack of this GP cannot firmly be proven, scaling may also appear in finite time or size windows and one can claim Griffiths effects only perhaps.

Otherwise the methodology seems to be sound, meets the expected standards and the presentation is clear. A minor point is that I have not found the definition of the exponent \alpha.

For the specialists one of the most noteworthy result is the appearance of such behavior, expected to arise in systems with quenched spatial heterogeneity. Here atomic motion can also alter or wash out the GP like behavior. Could the authors experience this "wash out" at certain experimental parameter values ?

In conclusion I highly recommend this important work to be published in Nature Communications, provided the above mentioned points are answered and clarified.

Reviewer #2 (Remarks to the Author):

The paper by Wintermantel et al. presents experimental results on and numerical simulations of a driven-dissipative system of Rydberg atoms. The authors find that the competition between facilitated excitation and spontaneous decay leads to dynamics that is reminiscent of epidemic spreading of diseases. They develop a numerical model of the susceptible-infected-susceptible type and find that the model reproduces the experimental results well. From this they conclude that rare active region effects play an important role in the dynamics of the system.
Overall, this is an interesting paper that is largely well-written and comprehensible to a broad audience (with a few exceptions I shall mention below). Having said that, the prominent mention of “epidemic spreading” in the title and in much of the first half of the paper gave me the impression that the authors were (over-)using that (obviously very timely!) concept as a major selling point. In fact, when trying to figure out which community would most benefit from the results of the paper (epidemiologists? Solid-state /statistical physicists? Cold-atom / Rydberg physicists?), it appeared to me that the paper does not really make an important contribution to epidemiology, nor does it

develop tools that could be used to that end in the future. By contrast, the use of the experimental and numerical tools described in the paper could well be seen as a powerful means of exploring the emergent heterogeneous networks mentioned in the paper.

This leaves the question of novelty: The underlying techniques of driven-dissipative Rydberg systems have been demonstrated in several experimental and theoretical studies cited by the authors. It seems to me that the main novelty, then, lies in the detailed analysis of the dynamics and the conclusions regarding the importance of the Griffiths phase (which, as it isn't a widely known concept, should be explained at a more intuitive level somewhere in the text). This is certainly a valid contribution to the field (both Rydberg physics and statistical physics), and in my view the authors should make this a little clearer.

In summary, the paper certainly merits publication and could potentially fulfil the criteria of Nature Communications, but I would ask the authors to consider the points made above, and the more detailed observations listed below.

P.3 Fig. 1 caption, last sentence: "..data not shown": Why is the data not shown? Surely, if they follow the theoretical curve, showing them would strengthen the authors' case?
p. 3, line 50: Why is no information given on the size of the density distribution?
p.4, line 52: "… around 8 seed excitations": I assume this is a Poissonian process, so 8 is the mean with a standard deviation around 2.8 ?
p-4, line 60: What about BBR-induced transfer to other Rydberg states? Would the fact that those processes are not included in the 2-level picture be likely to influence the observed dynamics?
p.4, line 60: "…. With an estimated rate…": Estimated from what? Why not measured?
p. 4, line 63: "… times t up to 2 ms": Is the trap on during that time?
p.4, line 64: "seemingly simple": Why "seemingly"? They are simple.
p. 4 line 77: "… lower than the estimated maximum number…" Have the authors checked that saturation effects on the detector can be ruled out? At what numbers would those become relevant?
p.4, line 80: "…gradual depletion…" What is that depletion due to? Loss of atoms from the trap due to Rydberg excitation (if so, why are Rydberg atoms lost and not recaptured?) or some other loss mechanism?
p. 5, Fig. 2 a: Above C=10, oscillations in the incidence rate are seen that are not discussed anywhere. Are those significant (and if not, why not), and what might be their origin?
p.5, line 83: How exactly is "incidence rate" defined? dC/dt?
p. 5, line 86: "…. Characterised by p=1" Would p>1 also be possible, and what would that mean?
p. 6, line 88: I think "tau" should be defined in a more prominent location, not in the parentheses here; on first and second reading I had difficulty finding the definition when tau came up later in the text.
p. 7 line 124: "… and the value of the two-dimensional Gaussian density distribution…" It is not clear what is meant by that ("value of the distribution")
p. 7 line 133: "… fixed number of … 8 seed excitations…" A fixed number is certainly not a good approximation to the (presumably) Poissonian process that actually occurs. Are the authors sure that this is a good approximation to make?
p. 8, line 157:) missing at the end of the sentence. This is the place I had difficulty locating the definition of tau earlier in the text.
p. 9, line 178: "over average": I think "above average" would be more common
p. 14, line 300: "…prepared at random positions in the gas…" While those will indeed be random, their probabilities will be proportional to the local density, won't it? Is that taken into account in the simulations, and do the authors think that this is relevant?
p. 14, line 316: "…eventually lost from the trap": Is that a reasonable assumption? Surely it could be checked experimentally?
p. 15 line 343 "kappa": probably a \ missing in Latex

Reviewer #3 (Remarks to the Author):

I enjoyed a lot reading the manuscript “Epidemic Growth and Griffiths effects on a Network of Excited Atoms”. It gives a fresh flavor to Rydberg physics and opens new perspectives for the future. The main topic of the paper, the dynamics of the epidemic growth in facilitated Rydberg systems is well described and analyzed. The match between experiment and theory is impressive. The experimental approach is conceptually clear and the theoretical model is well justified. The manuscript is very well written and free of any technical shortcomings. I recommend publication in Nature Communications, provided the authors consider to the following issues:
1. After reading the manuscript, I was wondering to which extend theory learned from experiment, or vice versa. I even asked myself, whether I would need the experiment to believe theory. Couldn’t the theoretical results just stand for themselves (given the fact that any real epidemic problem has different microscopic parameters as the Rydberg system)? So my devil’s advocate question to the authors is: Why should I spend one million dollar in a Rydberg gas experiment while I could buy a 4000 Dollar computer, which gives me the same results and the same understanding of epidemics?
2. In the discussion of the heterogeneous network, I was wondering, how close the hexagonal packing is realized in the experiment. Ref. 28 shows only one unit cell and it is well known, that the spatial correlation functions in blockaded Rydberg gases do not show pronounced oscillations. Do there is no real crystalline structure to expect in the experiment. To which extent does this affect the interpretation in terms of the network model?
3. Line 117 – 127: I find the explanation of how the network model is built difficult to follow. For instance, what are the weighted degrees s_i? What are the \alpha_i,j ?Maybe the authors can explain this in a bit more detail or they add a figure where, the model parameters are illustrated.
4. Exponent \alpha is not explained in the main text – it appears only in figure 2a in the supplementary material. The authors should explain it also in the main text.
5. I found the discussion of the Griffiths effects not clear enough. Not every reader is familiar with them and the authors should either spend more space to explain better, what they are, or they shall skip the last paragraph. At present, it reads almost like an appendix…

Response to the reviewer comments of the manuscript NCOMMS-20-30753-T

We greatly appreciate the careful consideration made by the referees in reviewing our manuscript and for their valuable comments.

We are very happy that the referees "enjoyed a lot reading the manuscript" and that they recognized it as "interesting paper that is largely well-written and comprehensible to a broad audience".

We especially appreciate the discussion about which communities would most benefit from this work, which we would like to generally address here. We see the main contribution as highlighting a controllable experimental system for exploring complex system dynamics on heterogeneous networks. This potentially fills a gap between empirical observations of real-world phenomena and abstract mathematical models. While it is hard to immediately see a direct application in (medical) epidemiology, we show the collective excitation dynamics of a strongly-interacting ultracold atomic gas can be understood in terms of a commonly used class of mathematical epidemiological models, which in turn opens up a controllable experimental system for exploring the predictions of these and other heterogeneous network models. We are therefore optimistic that this paper will be of value to a wide range of physicists, mathematicians, computer scientists and complex systems scientists. Associated discoveries (of more direct relevance in many-body physics and network science) concern the observation of sub-exponential scaling in the excitation growth and relaxation dynamics, the emergent heterogeneous character of the network and the appearance of rare region effects associated to a Griffiths phase which modifies the absorbing state phase transition.

Detailed responses to all the referee comments follow.

Point-by-point-response

Reviewer #1

R1.1:
Here we one arrives at a question how well the classical SIS model can describe an ultracold gas, which is a quantum system. I miss a discussion about it. Could the authors also determine the critical behavior perhaps ?
A1.1:
Quantum effects on the many-body dynamics (on the explored timescales) can be neglected in these experiments due to strong dephasing of the ground-Rydberg transition. Previous work has established that under these conditions the excitation dynamics can be very well described by classical rate models or effective Langevin equations derived from a quantum master equation describing the microscopic physics (see e.g. [Ref. 23]).
We added two sentences to quantify dephasing and timescales associated to coherent dynamics on p. 4 line 64 of the revised manuscript.

R1.2:

At line 189 "disorder-free absorbing state phase transition" is claimed for \kappa < \Gamma. What is the expectation for the universal critical point scaling : classical SIS, with quenched disorder or a quantum SIS with quenched disorder?

A1.2:

In the presence of strong dephasing, we expect that the disorder-free case corresponds to classical SIS on the emergent two-dimensional network.
In our experimental system, partially quenched disorder is always present and this makes the question of universality potentially even more interesting, as the critical point is replaced by a stretched Griffith region which is of current theoretical interest [Refs. 10,11,34].
We revised the text to mention the connection to the expected classical universality class on p. 7 line 123 along with the reference [Ref. 27]. We also clarify hat the present results concern a disordered system and that the mention of a disorder free transition at line 189 (now 208) was just for comparison.

R1.3:

The quantum contact process, which presumably shares the universality of quantum SIS has just recently been explored. I think a paragraph on this would be desirable to compare to the established literature.

A1.3:

Considering this work concerns the classical SIS model (see A1.1, 1.2) we prefer not to over-speculate concerning possible relations to the quantum contact process. However, we added a corresponding sentence in the Discussion section on p. 10 line 223 with references.

R1.4:

The work supports the conclusions and claims, based on numeric data of the excitation measurements and SIS simulations, however for claiming a real GP, defined in the thermodynamic limit, a system size independence should also been shown. In the lack of this GP cannot firmly be proven, scaling may also appear in finite time or size windows and one can claim Griffiths effects only perhaps.

A1.4:

It is true that our system, as any experimental or numerical system, can exhibit boundary effects due to finite system sizes. However, the experimental and numerical data clearly shows power-laws in the growth phase as well as the algebraic relaxation attributed to Griffith effects, without obvious finite size effects for the explored time scales.

We also emphasize that our discovery of the important role of heterogeneity and rare region effects in the emergent network description came as a welcome surprise and we expect that this work will trigger detailed studies of the Griffiths region to conclusively prove the Griffiths phase and corresponding scaling properties in the future (see opening paragraphs of this response). We added a sentence in the discussion about future possibilities to explore this phase diagram under even more controlled conditions (p. 10 line 215).

R1.5:

Otherwise the methodology seems to be sound, meets the expected standards and the presentation is clear. A minor point is that I have not found the definition of the exponent \alpha.

A1.5:
We would like to thank the referee for pointing out that the parameter α was only defined via equation (3) in the Methods section. This is now fixed, where we write in the main text that α refers to the power-law relaxation exponent and reference it to the Methods section (see on p. 5 caption of Fig. 2 and on p. 9 line 200).

R1.6:
For the specialists one of the most noteworthy result is the appearance of such behavior, expected to arise in systems with quenched spatial heterogeneity. Here atomic motion can also alter or wash out the GP like behavior. Could the authors experience this "wash out" at certain experimental parameter values ?
A1.6:
Yes, indeed the importance of quenched spatial disorder, even in the presence of atomic motion, is one of the surprising results. We elaborate on this starting on line 161 and further in Methods section starting line 257, where we point out that the velocity dependence of the facilitation constraint (with cutoff v_{LZ}) implies slow diffusion of the emergent network in phase space on a timescale $R_{fac}/v_{LZ} \gtrsim 2ms$. This is longer than the observations which were deliberately limited to minimize effects of the finite off-resonant seed excitation rate. We expect that the crossover from quenched to annealed or time-averaged disorder (depending on atomic motion) could be investigated in more detail in the future.

Reviewer #2

R2.1:
Having said that, the prominent mention of “epidemic spreading” in the title and in much of the first half of the paper gave me the impression that the authors were (over-)using that (obviously very timely!) concept as a major selling point. In fact, when trying to figure out which community would most benefit from the results of the paper (epidemiologists? Solid-state /statistical physicists? Cold-atom / Rydberg physicists?), it appeared to me that the paper does not really make an important contribution to epidemiology, nor does it develop tools that could be used to that end in the future. By contrast, the use of the experimental and numerical tools described in the paper could well be seen as a powerful means of exploring the emergent heterogeneous networks mentioned in the paper.
A2.1:
We share the view that our work represents a powerful means to explore emergent heterogeneous networks. Concerning our view of target audience, please see our general comments at the beginning of this response. We would just like to insist that the connection we find between the dynamics of a driven Rydberg gas and epidemic spreading is more than a selling point, supported by the appearance of sub-exponential growth described by the generalized growth model and the quantitative description of our experiment in terms of a heterogeneous SIS network model (widely known as epidemic models).

R2.2:
It seems to me that the main novelty, then, lies in the detailed analysis of the dynamics and the conclusions regarding the importance of the Griffiths phase (which, as it isn’t a widely known concept, should be explained at a more intuitive level somewhere in the text). This is

certainly a valid contribution to the field (both Rydberg physics and statistical physics), and in my view the authors should make this a little clearer.

A2.2:

Thank you for this suggestion. In the revised manuscript, we extended the paragraph starting on line 177 to explain at a more intuitive level the appearance of Griffiths effects.

R2.3:

P.3 Fig. 1 caption, last sentence: "..data not shown": Why is the data not shown? Surely, if they follow the theoretical curve, showing them would strengthen the authors' case?

A2.3:

Indeed, this was a bit confusing, since this paper does not concern the very late time dynamics [studied in Ref. 23], but is restricted to shorter timescales so that particle loss and spontaneous Rydberg excitation could largely be neglected (thus simplifying the data interpretation).

To avoid this confusion, we removed the relaxation part of the Fig. 1d.

R2.4:

p. 3, line 50: Why is no information given on the size of the density distribution?

A2.4:

We now mention the two Gaussian sigma values of the atom distribution on p. 4 line 51.

R2.5:

p.4, line 52: "… around 8 seed excitations": I assume this is a Poissonian process, so 8 is the mean with a standard deviation around 2.8 ?

A2.5:

The seed distribution is quite broad over the range from 0 - 16, presumably due to extra technical noise and drift of experimental parameters which could not be corrected for in the seed excitation process. We chose 8 seed excitations as a typical value, which is also verified by good agreement with the numerical simulations (which assume a constant initial number, see A2.17).

R2.6:

p-4, line 60: What about BBR-induced transfer to other Rydberg states? Would the fact that those processes are not included in the 2-level picture be likely to influence the observed dynamics?

A2.6:

We include BBR induced decay into our effective decay rate constant Γ (see p. 4 line 64). Beyond that, we expect that effects beyond the simple 2-level picture would modify the effective rates of some processes, namely the ratio of the facilitation to decay rate. However, the good agreement with a relatively simple heterogeneous network model as well as our previous experiments [Ref. 23] indicates that the detailed atomic physics (e.g. beyond the 2-level picture) do not drastically change the macroscopic emergent properties, e.g. exponents for sub-exponential growth and relaxation or critical properties, which can be understood as consequences of the dynamics on larger (coarse grained) scales.

We added an extra statement on the use of effective rates in the text lines 58-60.

R2.7:

p.4, line 60: "…. With an estimated rate…": Estimated from what? Why not measured?

A2.7:
The precise value of the off-resonant seed excitation rate is not too important as long as these events are relatively rare. We confirm that this is the case in a separate experiment without seed excitations where we see no dynamics for timescales up to ~1 ms (for the highest values of kappa), which is long compared to the initial growth dynamics. This is also verified from a calculation of the expected off-resonant seed excitation rate from the estimated laser parameters.
We updated the text to make this determination clearer on p. 4 lines 67-68.

R2.8:
p. 4, line 63: "… times t up to 2 ms": Is the trap on during that time?
A2.8:
Yes, the trap is on during that Rydberg excitation period. We clarified this in the experimental sequence in the Methods section on p. 11 line 229.

R2.9:
p.4, line 64: "seemingly simple": Why "seemingly"? They are simple.
A2.9:
True, our meaning here was not very precise. We adapted the text to "Although each atom is identical and its evolution is captured by these simple excitation rules,…" (p. 4 line 71).

R2.10:
p. 4 line 77: "… lower than the estimated maximum number…" Have the authors checked that saturation effects on the detector can be ruled out? At what numbers would those become relevant?
A2.10:
Yes, for these experiments we took care to avoid saturation effects. This is confirmed from the linearity of the conversion factor from MCP signal to Rydberg excitations. We calibrate this under similar conditions to the experiment by comparing the cumulative time-integrated MCP voltage with the total number of atoms lost from the trap (measured by absorption imaging) for different Rydberg laser exposure times. We do not observe any saturation effects up to at least 800 (instantaneous) Rydberg excitations.

R2.11:
p.4, line 80: "…gradual depletion…" What is that depletion due to? Loss of atoms from the trap due to Rydberg excitation (if so, why are Rydberg atoms lost and not recaptured?) or some other loss mechanism?
A2.11:
We experimentally observe that Rydberg-excited atoms are generally lost from the trap or no longer visible via absorption imaging [studied in Ref. 23]. We attribute this to several effects, for example thermal motion and the fact that Rydberg excitations experience an anti-trapping potential, which expels them from the trap on an estimated timescale that is comparable to the Rydberg lifetime. The presented experiments were restricted to timescales where depletion does not play an important role (we verify that the total atom number remains approximately constant for exposure times up to 2ms).

R2.12:
p. 5, Fig. 2 a: Above C=10, oscillations in the incidence rate are seen that are not discussed anywhere. Are those significant (and if not, why not), and what might be their origin?

A2.12:
We do not attribute significance to these small features as they does not appear to have a regular period and we cannot think of any physical origin for such oscillations. The data was taken in such a way that there should be no artificial correlations between datasets. Some visual appearance of correlations could arise due to the use of "cumulative incidences" which is a time integral of the "instantaneous incidence rate".

R2.13:
p.5, line 83: How exactly is “incidence rate” defined? dC/dt?
A2.13:
The incidence rate C' is defined as the instantaneous number of excitations divided by their lifetime τ: $C' = N_R/\tau = N_R \cdot 2\pi \cdot \Gamma$ (p. 6 line 90). Assuming a constant decay rate Γ, this can be interpreted as the rate at which new Rydberg excitations are created.

R2.14:
p. 5, line 86: “…. Characterised by p=1” Would p>1 also be possible, and what would that mean?
A2.14:
Super-exponential growth with $p > 1$ is in principle possible but would be quite unusual. The solution to the differential equation [Eq. 1 in the manuscript] with $p > 1$ blows up in finite time and is only physically well-defined up to a finite time before the blow-up occurs. [“Can growth be faster than exponential, and just how slow is the logarithm?”, J. Tolle, Math. Gaz., 87 (510) (2003), pp. 522-525]
To speculate a little, it might be possible to observe super-exponential growth in situations where the Rydberg excitation rate depends on a high power of the instantaneous number of Rydberg excitation. But this will hold only for a short time until other effects constrain the growth.

R2.15:
p. 6, line 88: I think “tau” should be defined in a more prominent location, not in the parentheses here; on first and second reading I had difficulty finding the definition when tau came up later in the text.
A2.15:
We added the definition of the lifetime \tau to the section on the experimental sequence and parameters (p. 4 line 63).

R2.16:
p. 7 line 124: “… and the value of the two-dimensional Gaussian density distribution…” It is not clear what is meant by that (“value of the distribution”)
A2.16:
We meant $n_{2d}(x_i, y_i)$. We changed the text to make it clearer (p. 7 line 133).

R2.17:
p. 7 line 133: “… fixed number of … 8 seed excitations…” A fixed number is certainly not a good approximation to the (presumably) Poissonian process that actually occurs. Are the authors sure that this is a good approximation to make?

A2.17:
We were able to reproduce the experimental data very well using a fixed number of 8 seed excitations. This is because the experimental data and the simulation data is averaged over many repetitions (A2.5).
Some fluctuations are implicitly included in the simulations, since we randomly assign seeds to nodes of the heterogeneous network, according to weights (A2.20) such that they can also fall in disconnect regions.

R2.18:
p. 8, line 157:) missing at the end of the sentence. This is the place I had difficulty locating the definition of tau earlier in the text.
A2.18:
Thank you. We added the missing ")" on p. 8 line 166.

R2.19:
p. 9, line 178: "over average": I think "above average" would be more common
A2.19:
We changed it to "above average" (p. 9 line 195).

R2.20:
p. 14, line 300: "…prepared at random positions in the gas…" While those will indeed be random, their probabilities will be proportional to the local density, won't it? Is that taken into account in the simulations, and do the authors think that this is relevant?
A2.20:
Yes, this is taken into account. We modified the text to make this sampling of the seeds clearer: "seed excitations randomly distributed among the nodes according to their weights N_i" (p. 7 line 142).

R2.21:
p. 14, line 316: "…eventually lost from the trap": Is that a reasonable assumption? Surely it could be checked experimentally?
A2.21:
The assumption that Rydberg-excited atoms are lost from the trap is consistent with previous experimental observations (see also A2.11).

R2.22:
p. 15 line 343 "kappa": probably a \ missing in Latex
A2.22:
We changed "kappa" to "facilitation rate" (see Supplementary Information p. 1).

Reviewer #3

R3.1:
1. After reading the manuscript, I was wondering to which extend theory learned from experiment, or vice versa. I even asked myself, whether I would need the experiment to believe theory. Couldn't the theoretical results just stand for themselves (given the fact that any real epidemic problem has different microscopic parameters as the Rydberg system)? So my devil's advocate question to the authors is: Why should I spend one million dollar in a

Rydberg gas experiment while I could buy a 4000 Dollar computer, which gives me the same results and the same understanding of epidemics?

A3.1:

For the results presented in this manuscript, the combination of experiment and theory went hand-in-hand and was essential for making several key discoveries:

Just like in an epidemic, it is impossible to accurately simulate the full dynamics of our experiment from first principles. So, the "art" is finding the best theoretical descriptions which capture the essential physics. Just to give two examples: Without the experimental observation of sub-exponential growth we probably would not have appreciated the strong effect of heterogeneity (which was a priori not obvious, given the thermal motion of the atoms, see the paragraph starting on line 161 in the manuscript). On the other hand, without the theoretical description of the dominant physics in terms of the heterogeneous network model we would not have recognized the importance of rare-region (Griffiths) effects in modifying the underlying phase transition (thereby explaining the emergence of power laws with varying power law exponents seen experimentally).

R3.2:

2. In the discussion of the heterogeneous network, I was wondering, how close the hexagonal packing is realized in the experiment. Ref. 28 shows only one unit cell and it is well known, that the spatial correlation functions in blockaded Rydberg gases do not show pronounced oscillations. Do there is no real crystalline structure to expect in the experiment. To which extent does this affect the interpretation in terms of the network model?

A3.2:

No, there is no real crystalline structure to be expected in the experiment. We chose the hexagonal structure since it allows the densest packing in two dimensions, but the excitation structures are always quite far from this densely packed configuration (paragraph starting on line 126). The emergent heterogeneous network is quite sparse, such that we can also achieve similar simulation results with a square lattice as long as the amount of heterogeneity $\epsilon(\kappa)$ is appropriately adjusted.

R3.3:

3. Line 117 – 127: I find the explanation of how the network model is built difficult to follow. For instance, what are the weighted degrees s_i? What are the \alpha_i,j ?Maybe the authors can explain this in a bit more detail or they add a figure where, the model parameters are illustrated.

A3.3:

The a_{ij} are the entries of the adjacency matrix (see p. 7 line 119 and line 126), which define which nodes in the network are connected by links. In the case of a triangular two-dimensional network for each node i, the a_{ij} values for the six nearest neighbor nodes j are 1, and the values to all other nodes are 0.

The weighted degree $s_i = \sum_j a_{ij} N_j$ (as defined on p. 7 line 136) of a node i is the sum over the other nodes j with the link weights a_{ij} times the node weights N_j. It is a proxy measure for the connectivity of each node and commonly used to characterize (heterogeneous) networks.

We added labels of a_{ij} and the node weights N_i into Fig. 1c to depict the network parameters better and added a little more detail to how the network is constructed starting from line 111.

R3.4:

4. Exponent \alpha is not explained in the main text – it appears only in figure 2a in the supplementary material. The authors should explain it also in the main text.

A3.4:

We would like to thank the referee for pointing out that the parameter \alpha was only defined via equation (3) in the Methods section. This is now fixed, where we write in the main text that \alpha refers to the power-law relaxation exponent and reference it to the Methods section (see on p. 5 caption of Fig. 2 and on p. 9 line 200).

R3.5:

5. I found the discussion of the Griffiths effects not clear enough. Not every reader is familiar with them and the authors should either spend more space to explain better, what they are, or they shall skip the last paragraph. At present, it reads almost like an appendix…

A3.5:

Thank you for this suggestion. In the revised manuscript, we extended the paragraph starting on line 177 to explain at a more intuitive level the appearance of Griffiths effects.

Reviewer #1 (Remarks to the Author):

The authors answered the questions well and corrected the manuscript. I can recommend it now for publication.

Reviewer #2 (Remarks to the Author):

The revised version of the manuscript takes into account my observations and those of the other referees. Overall, I think the paper is clearer and more readable now. I can recommend publication without any further corrections.

Reviewer #3 (Remarks to the Author):

The authors have adequately addressed my questions. The paper is ready for publication.

Point-to-point response to the reviewers' comments of the manuscript NCOMMS-20-30753-T

All three reviewers unanimously agree that it should be published as is. There are no further comments to address.

Reviewer #1 (Remarks to the Author):
The authors answered the questions well and corrected the manuscript. I can recommend it now for publication.

Answer:
We would like to thank the reviewer for the detailed comments and for helping in improving our manuscript.

Reviewer #2 (Remarks to the Author):
The revised version of the manuscript takes into account my observations and those of the other referees. Overall, I think the paper is clearer and more readable now. I can recommend publication without any further corrections.

Answer:
We would like to thank the reviewer for the detailed comments and for helping in improving our manuscript.

Reviewer #3 (Remarks to the Author):
The authors have adequately addressed my questions. The paper is ready for publication.

Answer:
We would like to thank the reviewer for the detailed comments and for helping in improving our manuscript.
